# Rolling away: a novel context-dependent escape behaviour discovered in ants

**DOI:** 10.1038/s41598-020-59954-9

**Published:** 2020-03-02

**Authors:** Donato A. Grasso, Daniele Giannetti, Cristina Castracani, Fiorenza A. Spotti, Alessandra Mori

**Affiliations:** Department of Chemistry, Life Sciences & Environmental Sustainability, University of Parma, Viale delle Scienze11/a, 43124 Parma, Italy

**Keywords:** Animal behaviour, Entomology, Behavioural ecology

## Abstract

For animals facing dangers, the best option to optimize costs and benefits of defence sometimes may be avoidance. Here we report the discovery of a peculiar strategy adopted by *Myrmecina graminicola*, a cryptic ant living in forest floor. Experiments showed that when disturbed these ants respond with immobility. However, upon perceiving disturbance but under specific inclinations of the substrate, they shift to an active escaping strategy: rolling away. This is a context-dependent behaviour adopted only in appropriate circumstances. During rolling, the ants assume a ball-like shape using antennae and hind legs to obtain an active movement along a stable trajectory. Finally, we assessed the adaptive value of this strategy measuring its effectiveness in defence against enemies. This is the first example of locomotion by rolling discovered in ants and one of the very few among animals, offering opportunities for multidisciplinary research on the adaptations and biomechanics underlying it.

## Introduction

The evolution of efficient systems of defence is one of the features behind the success of arthropods^1^. These animals are equipped with a variety of mechanical and chemical weapons as well as behavioural strategies enabling them to contrast dangers^1–3^. Species that form aggregations or present a higher level of social organization may rely on both individual strategies and collective reaction to danger mediated by alarm communication^4^. In any case, the best defence is not always withstand attack and/or counterattack the enemy. Sometimes, depending on ecological and morpho-functional constraints, the best option to optimize costs and benefits of defence is avoidance^5,6^. Ants offer remarkable examples of both social and individual defensive strategies^7^. Under specific ecological conditions, some species evolved morpho-functional and behavioural adaptations allowing a sudden departure from the threat. Jumping, as in the case of *Harpegnathos saltator* and other ants, is one of the most obvious system to get away rapidly^8^. This may be achieved also in a non-conventional manner such as in *Odontomachus* trap-jaw ants that snap their mandibles on a hard surface to be catapulted away from predators^9^. Another peculiar defensive jumping is adopted by the Malagasy cliff dwelling ant *Malagidris sofina* whose workers defend their nest from intruders by dropping off the cliff face while clinging to invaders^10^. A striking technique used by ants to rapidly avoid enemies is adopted by workers of the canopy dwelling ant *Cephalotes atratus* that drop down from trees and glide following a spiral path that bring them safe again on the trunk of the home tree^11^.

In the present study, we report a novel defensive behaviour recorded in ants and based on a peculiar escaping strategy in presence of a potential source of danger: curling into a ball and rolling away. The studied ant is *Myrmecina graminicola*, a cryptic species with West Palaearctic and Mediterranean distribution found in leaf litter, generally in forested areas or grasslands. Colonies are very difficult to find being inconspicuous and occurring in very heterogeneous environments (soil with or without coverings, among rocks, in twigs on the ground or in rotten wood). Workers are quite elusive and seldom observed in the field being small (~3 mm long), dark coloured, slow moving and scavenging or preying on small invertebrates. Anecdotal descriptions report that upon disturbance they tend to freeze or curl their body as a defensive system, sometimes referred to as a case of thanatosis as it happens in other arthropods^12,13^. However, in the field we had the chance to observe some encounters with predators (e.g. Agelenidae spiders) and other ants (e.g. *Temnothorax* spp.) that attacked *Myrmecina* workers. In these occasions, *Myrmecina* ants reacted in a dynamic way: upon enemy harassment, they moved forward, curled into a ball and rolled away exploiting the slopes of the extremely uneven terrain. Starting from these occasional observations we decided to deepen the study of what looked like an exceptional behaviour since to date moving by rolling is reported for only a very few animals^14–17^. We analysed the rolling behaviour of *M. graminicola* showing its features and adaptive value. We also tested the hypothesis that this is a context-dependent defensive strategy adopted only when the escaping technique is effective and appropriate rather than being a mere by-product of curling the body whenever a danger is perceived.

## Results

### Reactions to disturbance on horizontal plane (Experiment 1)

As first step of our investigation, we analysed ant reactions to mechanical disturbance perceived while moving on a horizontal plane. Significantly different reactions (freezing, curling, and walking) were recorded upon receiving slight and strong vibrations (Chi-square test: *χ*^2^_2_ = 97.5, *P* < 0.001), as well as slight and strong tapping on the gaster (Chi-square test: *χ*^2^_2_ = 111.6, *P* < 0.001). In particular, the analysis of Standardized Residuals (see Supplementary Information and Supplementary Table S1) showed that upon receiving both slight mechanical stimuli, the most commonly adopted behaviour was sudden freezing. However, after strong disturbance (making the ants to lose the contact with the substrate) most ants assumed a curled (ball-like) position. This is probably a mechanical protection induced by the sudden detachment from the substrate caused by a dramatic distress situation.

### Walking ability on inclined planes and reaction to disturbance (Experiment 2)

Tests on the ability of the ants to move on planes at different inclinations (from 0° to 45°, every 5°; 90° was also tested) showed that none of them rolled down or showed any troubles to walk along the whole path for each of the considered gradients, even at vertical slope. This was preliminary to study ant reactions to disturbance offered on the same planes.

**Figure 1:**
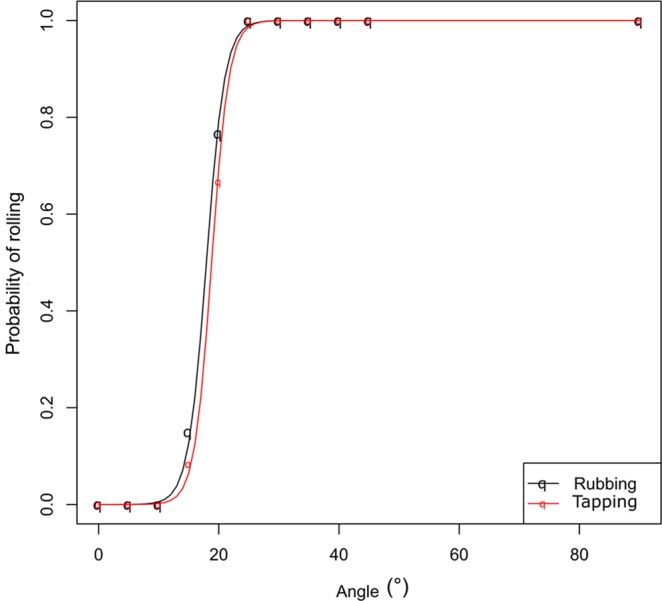
Logistic Regression Model showing the probability of rolling at different inclinations of the plane upon receiving mechanical stimulations that do not cause loss of contact with the substrate. Line and symbols (q) in black: rolling induced by substrate rubbing behind the walking ant; line and symbols (q) in red: rolling induced by a slight tapping on the experimental device.

In this case, ants reacted to vibrational stimuli (slight tapping or rubbing on the substrate) in a similar way, showing rolling behaviour only on inclined planes. It is worth noting that both experimental stimuli did not cause any loss of contact with the substrate by the ants. We recorded a highly significant effect of the inclination of the path on the probability of rolling upon perceiving the stimulus (Logistic Regression Model, rubbing: *χ*^2^_1,10_ = 752.3, *P* < 0.001; tapping: *χ*^2^_1,10_ = 767.3, *P* < 0.001, n = 60 for each angle of each treatment). In particular, for both vibrational stimuli, a higher than 10° inclination should be reached to have a non-zero probability of assuming a curled (ball-like) position and rolling down. Critical slope for a 100% probability of occurrence of rolling behaviour resulted 25° in both cases (Fig. 1). These results mean that: (a) both the “stimulus” and the “inclination” taken alone do not induce any rolling behaviour; (b) there is a threshold slope of the plane (10°) starting from which on the behaviour is triggered and is presumably more effective.

### Analysis of different phases of body curling and active rolling on inclined plane (Experiment 3)

**Figure 2:**
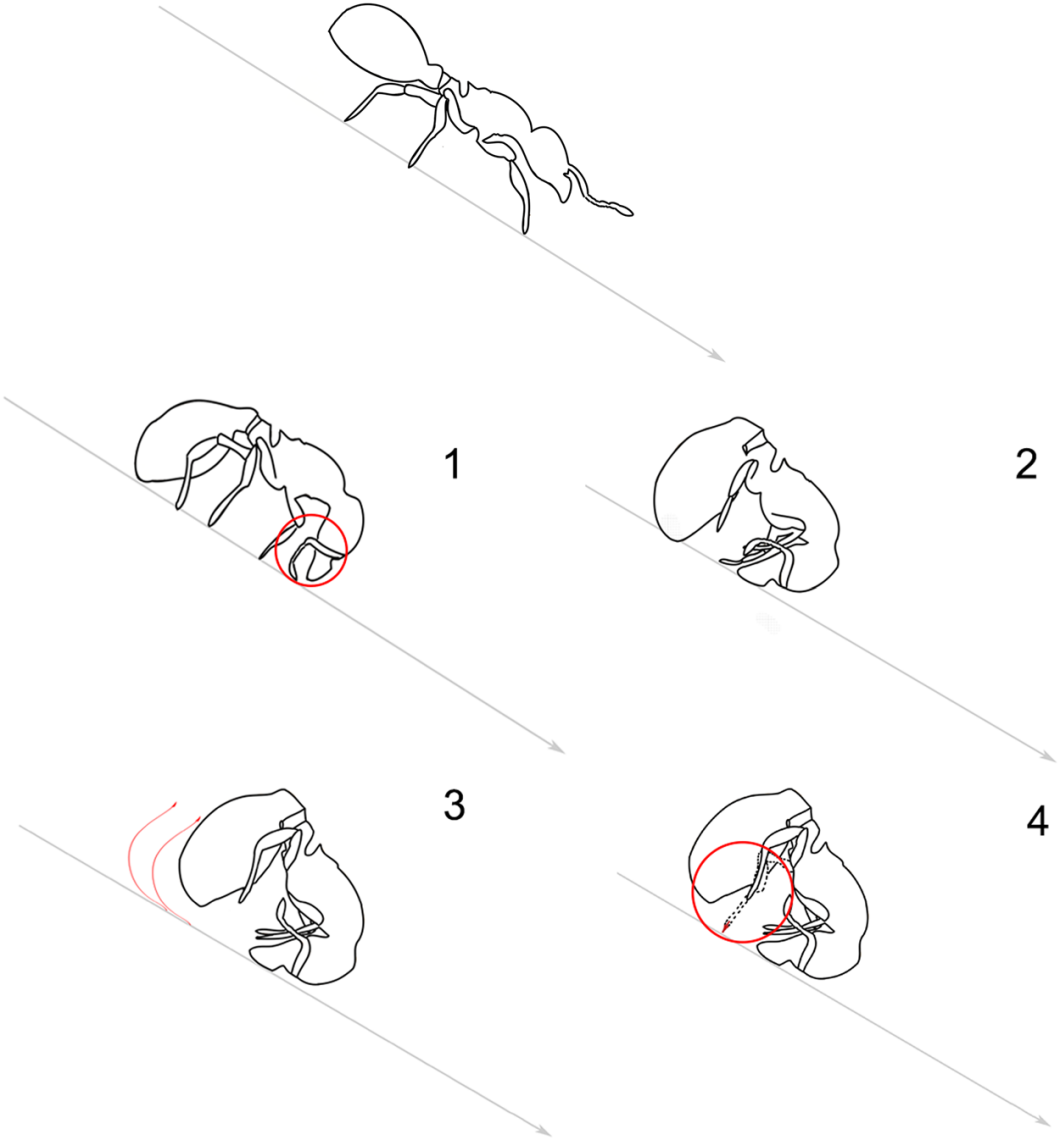
Schematic description of the different steps of *M. graminicola* rolling behaviour on inclined plane. Top: normal walking position; (1) antennae and mandibles are used as support to prepare for rolling; (2) head and tip of the gaster move toward each other and legs go up; (3) the ant starts rolling; (4) hind legs extension as possible system to further pushing the ant.

The accurate analysis of macro videos (60 fps) both frame-by-frame and played at slow-motion (25% reduced speed) allowed a fine description of the different steps of the rolling movement under specific conditions (25° inclined plane, robbing the substrate behind the ant without touching it) (Supplementary Video S1). They are summarized as follows (Fig. 2). *Step 1*: the antennae move backwards along the head soon after the tip of the forceps touches the substrate (average reaction time = 0.128 ± 0.02 s, n = 10), then the distal portion of the funiculus is put in contact with the substrate. *Step 2*: the head and the tip of the gaster move toward each other and the legs go up. At this stage, the head (mandibles and antennae) and the gaster represent the only body parts in contact with the substrate and the ant assumes a curved position. *Step 3*: as step 2 is proceeding, the ant body assumes a curled shape and starts rolling down; the latency to roll (from the touch of forceps on the ground to the beginning of rolling) takes in average 0.53 ± 0.08 s (n = 10). *Step 4*: before the first turn is completed, the ant in addition to using the antennae as a sort of “arms” can extend the hind legs; we suggest that this last movement may eventually act as a kick to further pushing the ant. Under these experimental conditions, ants walked in average at 0.47 ± 0.03 cm/s (n = 12) while during rolling they reached in average a linear speed of 39.7 ± 1.5 cm/s (n = 20).

### Duration of the ball-like position (Experiment 4)

**Figure 3:**
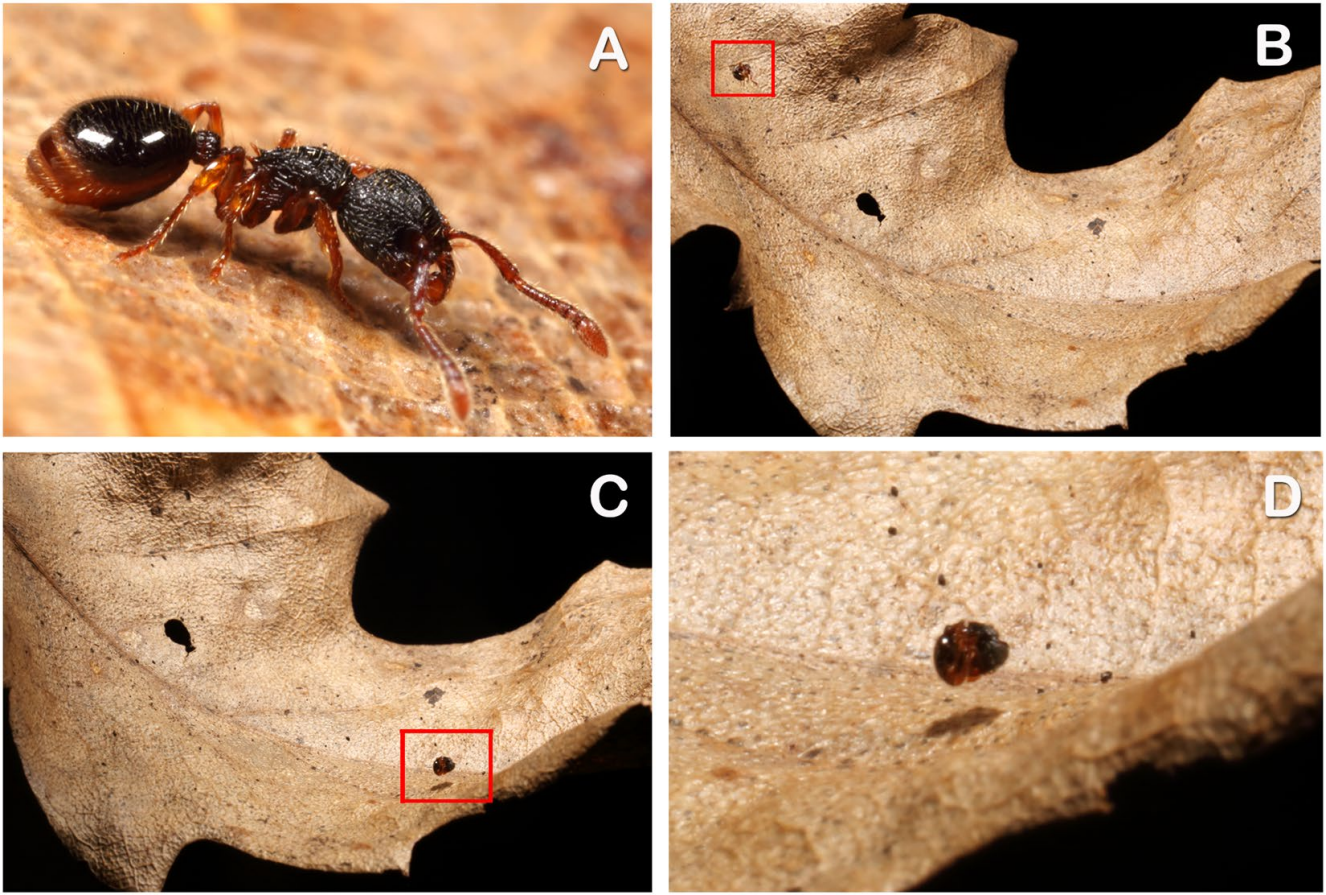
Rolling behaviour of *M. graminicola* on a natural substrate. (**A**) Worker walking on a leaf before rolling. (**B**) Rolling starts upon receiving a disturbance (in this case substrate rubbing). (**C**) The leaf acts as a springboard making the rolling ant (inside the red square) bouncing. (**D**) Close up of the bouncing ant showing the ball-like shape assumed by the curled body. The ant is ~3 mm long.

**Figure 4:**
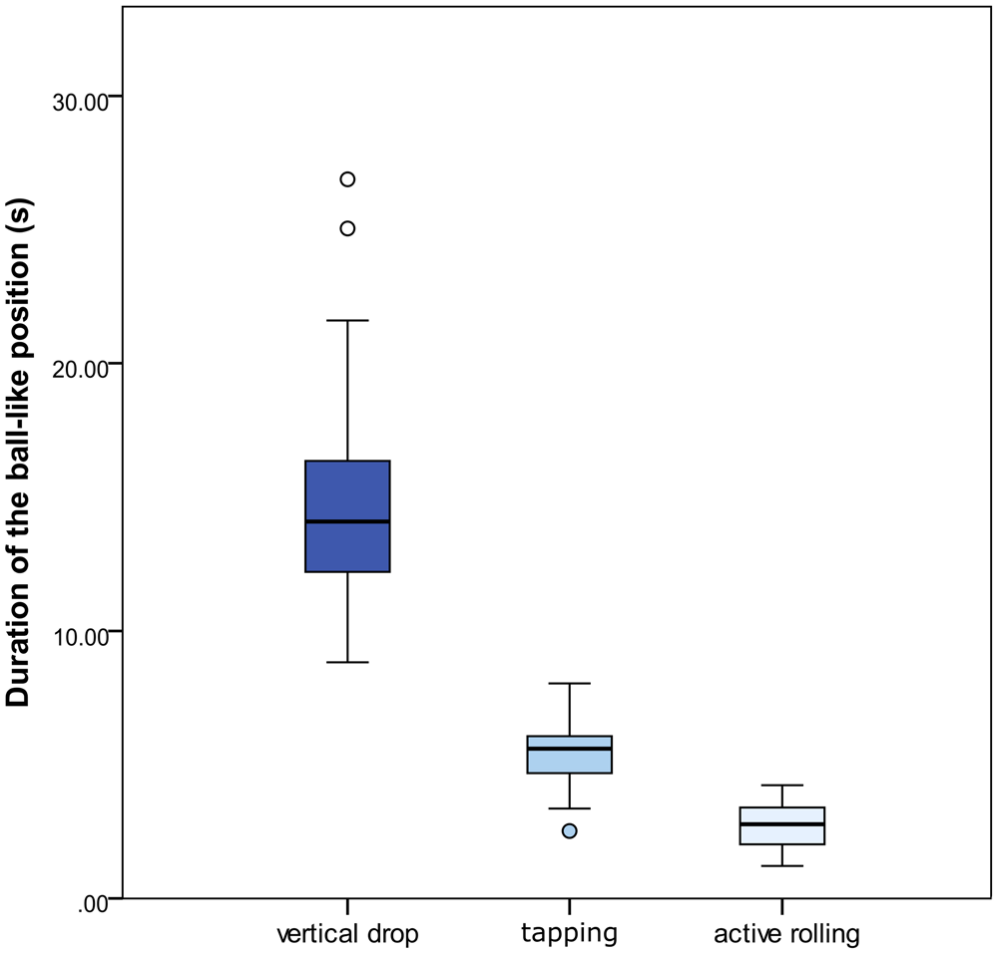
Box-and-whisker plot of the duration of the ball-like position kept by the ants upon receiving different distress stimuli causing the loss of contact with the substrate. Horizontal line within each box represents the median value. Outliers (o) are reported. Highly significant differences were recorded among treatments (Kruskal-Wallis test, *H*_2_ = 156.256, *P* < 0.001; Pairwise multiple comparisons, *P* < 0.001 for all comparisons, n = 60 for each treatment).

Once the movement is finished, the ant keeps the ball-like position assumed during rolling (Fig. 3) for a few seconds before resuming walking. However, the duration of this position is different according to the cause of falling (Kruskal-Wallis test: *H*_2_ = 156.256, *P* < 0.001, Fig. 4) being higher if the events causing a loss of contact with the substrate are more dramatic. In particular, after a strong tap on the gaster or a vertical drop (that may simulate unexpected and strong distress situations) the ants keep a ball-like shape for a longer time (respectively, mdn 5.6 s, min-max: 2.55–8.04 s, and mdn 14.09 s, min-max: 8.83–26.88 s, in both cases n = 60) than after an active rolling (mdn 2.77 s, min-max: 1.21–4.3 s, n = 60) (Pairwise multiple comparisons adjusted: *P* < 0.001 for all comparisons). Hence, the ants are prone to stay curled for a longer time if the cause of falling is a sudden and mechanical factor rather than during active escaping from a danger by rolling (Supplementary Video S1 and S2).

### Rolling on natural substrates (Experiment 5)

**Figure 5:**
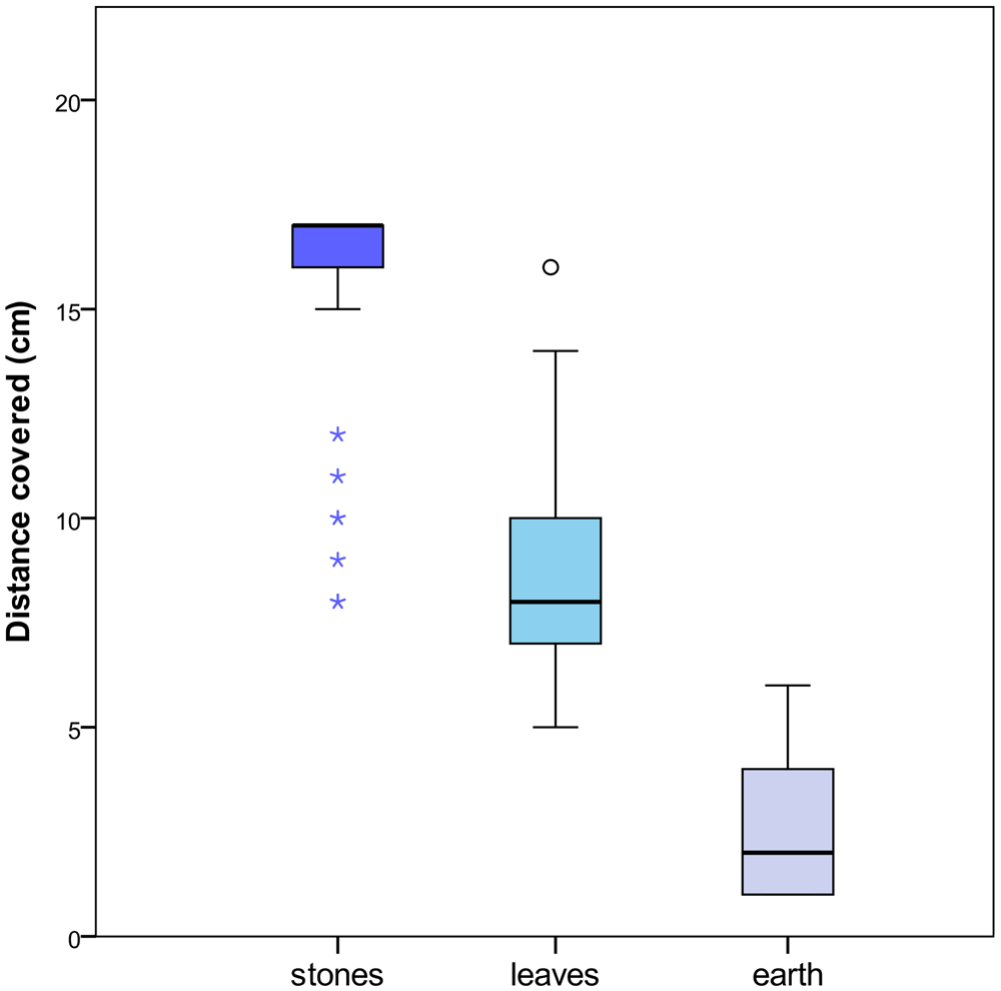
Box-and-whisker plot of the distance covered after rolling on different natural substrates. Horizontal line within each box represents the median value. Outliers (o) and extreme values (*) are reported. Highly significant differences were recorded among treatments (Kruskal-Wallis test, *H*_2_ = 154.920, *P* < 0.001; Pairwise multiple comparisons, *P* < 0.001 for all comparisons; n = 60 for each treatment).

Ants showed the rolling behaviour also on natural substrates (earth, leaves and stones) (Fig. 3). However, significant differences were recorded in the distances reached after rolling (Kruskal-Wallis test: *H*_2_ = 154.920, *P* < 0.001, Fig. 5). In particular, on a layer of earth the ants rolled for only a few cm (mdn 2, min-max: 1-6, n = 60), while on leaves and stones they reached longer distances (respectively: mdn 8 cm, min-max: 5–16 cm, mdn 17 cm, min-max: 8–17 cm, n = 60 in both cases) before resuming walking or going out of sight (vanishing point) under slits among stones or leaves. Pairwise multiple comparisons adjusted showed highly significant differences in the comparisons among all substrates (*P* < 0.001 for all comparisons).

### Reaction to aggression (Experiment 6)

**Figure 6:**
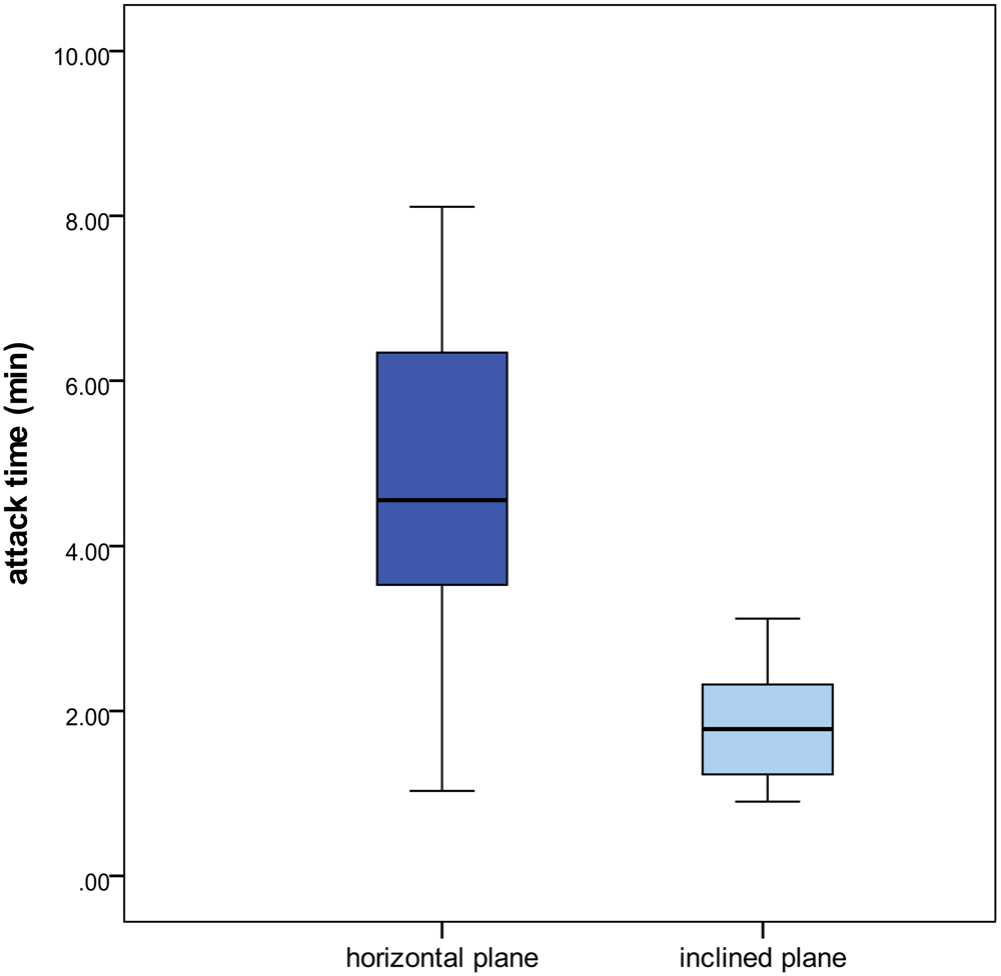
Box-and-whisker plot of the time spent on attack during aggression tests (*M. graminicola* vs *T. unifasciatus*) carried out on horizontal and inclined planes. Horizontal line within each box represents the median value. Highly significant differences were recorded between treatments (Mann-Whitney U test, *U* = 45, *P* < 0.001, n = 30 for each treatment).

Aggression tests (carried out on horizontal or inclined plane) allowed quantifying the adaptive value as defensive system of this peculiar escape system under the attack by an enemy (in this case the competitor ant *Temnothorax unifasciatus*). Attacks were usually started by *T. unifasciatus* (93.3% on horizontal and 96.7% on inclined planes) while *M. graminicola* assumed a rather passive habit seldom reacting to the opponent. On the horizontal plane, in only 3 cases (10%) *Myrmecina* ants assumed a curled position, but this occurred only after having been picked up (and so detached by the substrate) by the opponent. On the inclined plane, 100% of rolling was recorded giving the ants a significant chance of escaping the attacks and reducing the damages of fighting. In fact, the aggressions suffered by *Myrmecina* ants resulted significantly less harmful when they were allowed to roll down. Total time spent on fighting was lower on inclined (mdn 1.78 min, min-max: 0.9–3.12 min) than on horizontal plane (mdn 4.55 min, min-max: 1.03–8.11 min) (Mann-Whitney *U* test: *U* = 45, *P* < 0.001) (Fig. 6), as well as the percentage of *Myrmecina* ants suffering injuries/death (10% on inclined plane, 63% on horizontal plane) (Chi-square test: *χ*^2^_1_ = 18.37, *P* < 0.001). This is probably due to the effectiveness of rolling as a system to disentangle from the attacker bites during or soon after the rolling phase (77% of cases) (Chi-square test: *χ*^2^_1_ = 8.53, *P* = 0.003).

## Discussion

In the present work, we report the discovery of a novel defensive behaviour adopted by ants: escaping by active rolling. In the struggle for life, defence from the attacks of enemies is a primary strategy. This is also true in the inconspicuous world of forest leaf litter or other microcosms. If you are not equipped with powerful (individual or social) weapons to counterattack enemy harassment, the best way to survive may be become undetectable or to escape. To do this, *Myrmecina graminicola* (a tiny and elusive ant living in leaf litter of deciduous forested areas) evolved a peculiar system: once detected a potential danger these ants may adopt freezing or, alternatively, they curl the body, form a ball and actively roll along slopes on the ground if present. This is an example of how, in specific ecological contexts, the best option to optimize costs and benefits of defence may be avoidance, that is reducing the probability of encounter/detection by an enemy or enhancing survival after detection^6^. Ants, in addition to adopting systems of social defence mediated by alarm/recruitment signals, may rely on a variety of individual strategies similar to those of solitary animals. Some species evolved avoidance strategies based on immobility and others on fast and/or tortuous movements, gliding from trees or jumping away^8,9,18,19^.

Escape by rolling is not an obvious way of defence, so the peculiar locomotion described here for *M. graminicola* is a very special case. In fact, to date only a few animals are reported to adopt it^20^. The most studied are some desert spiders of the genus *Carparachne* rolling down along smooth sand dunes^14^, the caterpillars of the moth *Pleuroptya ruralis* that form a backwardly rolling wheel to escape disturbance^17^, and the intertidal stomatopod *Nannosquilla decemspinosa* that prefers to flip its body across the wet sand when there is no water to swim^15,21^. Curling into a ball to be transported to a safer place by moving water has been recently described also in chitons^22^. Among vertebrates, only one case has been documented so far, the salamander *Hydromantes platycephalus* whose action consists of body and tail coiling, limb tucking followed by passive rolling along slopes^16^. There are several anatomical and physical constraints to wheeling movements as a general mode of moving in animals^23^. However, constraints are probably less severe if, rather than using body parts as wheels, the agent is able to become itself a wheel or a ball-shaped object that can roll down along slopes, jump and rebound on the ground to overcome obstacles found on the path or exploit the springboard effect offered by objects present in the environment. This is exactly what *M. graminicola* ants do in a very heterogeneous habitat such as the leaf litter. We verified that, for a rolling ant, rocks and leaves offer a substrate more suitable than earth to reach longer distances before disappearing underneath the ground materials or resuming walking to escape. Earth pebbles probably provide more friction and obstacles to a small rolling object, while stones and leaves offer harder, smoother and curved surfaces that ensure a springboard effect and make the rolling ant to act as a sort of bouncing ball. In any case, earth grounds may offer the possibility of an effective hiding for ants fleeing or freezing, being *M. graminicola* small, dark coloured and presumably quite cryptic for vertebrates or arthropods hunting on sight. Similar behaviours have been reported for other cryptic ants (e.g. *Basiceros manni*) using slow movements or immobility that, in association with morphological elements, may favour camouflage when a potential danger is approaching^18^.

The rolling movement of *M. graminicola* is very rapid and requires a series of adjustment (for example the antennae are used as a sort of arms) to complete body morphing and assume an efficient ball-like position allowing a stable trajectory. All happens in about half a second from the moment the distress stimulus is offered. As in other rolling invertebrates, the linear speed reached by these ants is high (about 40 cm/s in our experimental conditions). This is far superior to that obtained by walking (about 0.5 cm/s in the same experiments) or by normal locomotion in other arthropods including potential predators (e.g. 2.63 cm/s, agelenid spiders; 5.66 cm/s, carabid beetles) and similar sized ants (e.g. 1.17 cm/s, *Tetramorium caespitum*)^14,15,17,20,23,24^. Rapid extension of the hind legs (a sort of kick) as rolling starts probably contributes to these performances. Importantly, we showed that the escape strategy by rolling of *M. graminicola* is context-dependent, being actively adopted only after reaching critical slope threshold (>10°) or after strong physical disturbance making them losing the contact with the substrate. We also verified that after falling the ants stay curled for a longer time if the cause of disturbance is a sudden and unexpected mechanical factor (strong tap on the gaster or a vertical drop) rather than during active escape by rolling. This may reflect the defensive strategy adopted by *M. graminicola*. In fact, rolling along a gradient after perceiving a potential danger is only the first step of the avoidance strategy being a rapid fleeing in search for a safe place the second one (see Supplementary Video S1 and S2). This is in accordance with other context-dependent strategies reported in different aspects of ant life, including individual/collective responses to dangerous situations^5,25–27^.

The rolling behaviour of *M. graminicola* is an ecologically appropriate and effective escape strategy. In fact, we evaluated the adaptive value of this strategy measuring its effectiveness as a defensive system against enemies. Rolling gives the ants a major chance of safe escaping the attacks of other ant opponents, reducing significantly injuries and death.

Questions arise on the morpho-functional and behavioural adaptations making this kind of rolling possible. For example, we can speculate that *M. graminicola* evolved some peculiar shape and manoeuvrability of body parts to form an effective rolling object, as well as a special arrangement of the neurosensory and motor equipment allowing these context-dependent performances as reported in jumping ants and trap-jaw ants^28–31^. These aspects would deserve further investigation since these ants represent a new study model for possible application in biomechanics and robotics of rolling agents^17,20,32^.

Finally, open questions remain on the constraints that have made this behaviour unique (or at least limited its spread) among the 13.200 ant species described so far, as well as on the evolutionary pathway that led from walking or other pre-existing movements (e.g. curled pupal position assumed during social carrying) to fleeing away “just like a rolling stone”.

## Methods

### Colony collection and maintenance

Three colonies of *M. graminicola* were collected from natural nests (inside oak galls fallen on the ground) located in a leaf litter of a wooden area in Fornoli (MS) (Tuscany, Italy). Colony fragments (about 40–50 workers, a queen and some brood each) together with their original gall nests were housed in plastic arenas (10 × 10 × 3 cm) with a plaster floor on which a 2 cm layer of sifted organic material taken from the field was placed. Laboratory conditions were controlled (T 20°/23 °C, RH 50/60%) with a 12 h light-dark cycle. Colonies were fed on a mixed solution of water, sugar and mealworms fragments. Small pieces of pumpkin seeds were also provided.

### Experimental set-up

For each trial of each experiment 60 replicates (except for experiment 3 and 6, see below) were conducted and single ants (20 from each colony) were tested only once for each treatment. Trials were carried out randomly from 10.00 a.m. to 4 p.m. To study ant reactions to a potential danger (a simulation of predator/enemy approaching or other distress situations causing ground vibrations or loss of contact with the substrate) we offered different kind of stimuli as described below.

### Reactions to disturbance on horizontal plane (Experiment 1)

The ants were introduced into experimental arenas (10 × 10 cm, floor covered by filter paper) and after 5 s, their possible reactions (freezing, walking, body curling into a ball) to the following stimuli were recorded:

1. Slight tapping of the horizontal plane by a plastic stick (n = 60).
2. Strong tapping of the horizontal plane by a plastic stick making the ant bounce (n = 60).
3. Slight tapping on the ant gaster by the tip of an entomological forceps (n = 60).
4. Strong tapping on the gaster by the tip of an entomological forceps causing the ant to lose contact with the substrate (n = 60).

### Walking ability on inclined planes and reaction to disturbance (Experiment 2)

The effect of slopes on the walking ability of the ants was tested allowing single individuals to freely move on inclinable plastic plane (5 × 17 cm) covered by filter paper (changed after every trial) with a 3 cm horizontal platform on which the ants were released. The number of ants that covered the entire path length and those eventually falling down (within 5 min experimental time) were recorded. Different inclinations of the plane were tested, from horizontal (0°) to 45° (every 5°). Vertical inclination (90°) was also tested (n = 60 for each inclination).

This was preliminary to a second set of experiments aimed to test how the ants behave upon receiving mechanical stimuli without being directly touched while moving along inclined planes (experimental device and gradients as above). As stimuli, we offered different slight vibrations on the ground that did not cause the ants losing the contact with the substrate:

- Single slight tap with a plastic stick at the base of the experimental device (n = 60).
- Slight substrate rubbing using an entomological forceps just behind the walking ant (avoiding any contacts with the ant body) (n = 60) (Supplementary Video S1).

In both cases, stimuli were offered ~5 sec after the ant release.

### Analysis of different phases of body curling and active rolling on inclined plane (Experiment 3)

Using the same conditions and experimental device as described in Exp. 2b, we recorded and analysed 10 videos (each involving a different individual) for a fine analysis of ant movements. Here and for all further experiments on inclined planes, a gradient of 25° was chosen because this was the critical slope for a 100% probability of rolling by the ants (see Fig. 1) (n = 10). For a better understanding and description of the different movements during rolling, we made a frame-by-frame analysis on macro videos. This allowed a better visualization of the sequence of movements of the body parts. Then we played the videos at slow motion (25% reduced speed), in order to observe and better describe the whole process.

Recordings were performed at 60 fps (progressive) using Canon 80D and 24 fps (progressive) using Blackmagic Cinema Camera Pocket with Metabones Speedbooster Adapter; Lens: Canon 100 mm Macro f/2, 8 IF USM, Sigma 24-105 f/4 DG OS HSM, Tamron 85 f/1, 8 Di VC USD; Tripods: Manfrotto 055X Pro with head Manfrotto 808 RC4, Manfrotto MK290 Dual with head 128RC and Edelkrone Slider One. To illuminate the experimental area, a set of three cold light lamps (85 W) was used with soft box (60 × 60 cm). For video editing and digital crop Final Cut Pro X software was used.

The clips (60 fps progressive) were analysed frame-by-frame by Kinovea software (www.kinovea.org) and a sequence of movements was described as a general pattern under those specific experimental conditions. The “reaction time” was measured as the time from the frame at which the forceps touched the substrate to the frame at which the antennae started moving backwards to prepare rolling. The “latency to roll” was measured as the time from the frame at which the forceps touched the substrate to the frame at which the ant started rolling downwards.

To measure the linear speed (distance/time) of the rolling ants (approximated to points), we analysed 20 videos each involving a different ant. Rolling was induced as in Exp. 2b. However, in this case to evaluate the distance covered from the starting point to the end of the rolling path the plane was covered by a graph paper. The duration of the movement was measured by Kinovea software (n = 20). By the same methods, we also evaluated the walking speed of the ants (n = 12) just before being offered the stimulus that induced rolling.

### Duration of the ball-like position (Experiment 4)

We measured how long the ants (singly tested in the same arena as in Experiment 1) kept the ball-like position upon receiving three different strong distress stimulations. (A) Tapping the ants from behind with the tips of a forceps making them loosing contact with the substrate; (B) vertical drop from a height of 10 cm (the ants were slightly pushed down from a horizontal plane); (C) active rolling on a 25° inclined plane after substrate rubbing with forceps (as in Exp. 2b) (n = 60 for each treatment).

### Rolling on natural substrates (Experiment 5)

In this set of experiments, the rolling behaviour on natural substrates (leaves, earth and stones) was evaluated using materials collected from the field area where colonies were found. Leaves, earth or stones were placed on an inclined plane (25°) on a wooden support (15 × 7 × 13 cm). The ants were introduced on the device and singly tested. Disturbance was obtained by substrate rubbing with an entomological forceps just behind the walking ant as in Exp. 2b. For each trial, we recorded if the ant rolled down and the distance reached before resuming a normal walking or before going out of sight (vanishing point) in the case of leaves and stones (n = 60 for each treatment).

### Reaction to aggression (Experiment 6)

Reaction to aggression was tested and the effectiveness of rolling as a defensive strategy evaluated. Aggression tests with other ants (*Temnothorax unifasciatus* collected in the same area of *M. graminicola*), were carried out in fighting boxes (4 × 4 × 2 cm, floor covered by filter paper) where dyads were introduced. Tests (experimental time 10 min) were conducted on both horizontal and inclined planes (25°, 20 cm long). A paper bulkhead separating the ants was removed 5 min after the ants were introduced into the box. In experiments on inclined plane, a second bulkhead (representing one of the wall of the box) was removed allowing the ants eventually to roll down to a second arena (10 × 10 × 2 cm). For this experiment, 30 replicates (10 ants from each colony) of each kind of aggression test (horizontal/inclined) were carried out. Ants were tested only once for each treatment.

A general description of the ants’ behaviour and the following occurrences were recorded:

- Ant initiating the attack;
- Curling of the body into a ball;
- Rolling behaviour (only for inclined plane);
- Defensive effectiveness of rolling (only for inclined plane), measured as the percentage of *Myrmecina* ants disentangling from the *Temnothorax* attack during or at the end of the rolling;
- Number of injured or dead ants;
- Attack time (total time spent on aggression).

### Statistical analysis

Chi-square test (2 × 3 tab) and following Analysis of Standardized Residuals were used to evaluate data from Exp. 1 (software: IBM SPSS Statistics, Italian version 24). A Logistic Regression Model was used for analysis of data from Exp. 2 (software: R version 3.3.1, www.R-project.org). Kruskal-Wallis test followed by pairwise multiple comparisons (Bonferroni correction) were used for analysis of data from Exp. 4 and Exp. 5, Mann-Whitney U test and Chi square test for Exp. 6 (software: IBM SPSS Statistics, Italian version 24).

Throughout the text, average values are reported with ±standard errors, while median values (mdn) are reported with minimum-maximum (min-max) range values.

### Animal use and welfare

All experiments reported in the present research were performed in accordance with relevant guidelines and regulations on animal use.

## Supplementary information

Supplementary information.

Supplementary information S2.

Supplementary information S3.

Supplementary information S4.

## Data Availability

All data generated or analysed during this study are included in this published article (and its Supplementary Information files).
