# Supplementary material for: Rolling away: a novel context-dependent escape behaviour discovered in ants
Source: Sci Rep. 2020 Mar 2;10:3784. doi: 10.1038/s41598-020-59954-9 (PMC7051971; doi:10.1038/s41598-020-59954-9)
Supplement: Supplementary file 2 — Supplementary information S2. [file 41598_2020_59954_MOESM2_ESM.docx]

**Supplementary Information for:**

**Rolling away: a novel context dependent escape behaviour discovered in ants**

Donato A. Grasso*, Daniele Giannetti, Cristina Castracani, Fiorenza A. Spotti, Alessandra Mori

Department of Chemistry, Life Sciences and Environmental Sustainability, University of Parma, Viale delle Scienze 11/a, 43124 Parma (Italy)

* Corresponding Author, D.A. Grasso e-mail: [donato.grasso@unipr.it](mailto:donato.grasso@unipr.it)

Co-Authors e-mail:

D. Giannetti: [daniele.giannetti@unipr.it](mailto:daniele.giannetti@unipr.it) , C. Castracani: [cristina.castracani@unipr.it](mailto:cristina.castracani@unipr.it)

F. A. Spotti: [fiorenzaaugusta.spotti@unipr.it](mailto:fiorenzaaugusta.spotti@unipr.it), A. Mori: [alessandra.mori@unipr.it](mailto:alessandra.mori@unipr.it)

**Extended description of analyses and results of Experiment 1 (Reactions to disturbance on horizontal plane)**

Difference in the frequency of responses to different disturbances was evaluated on the horizontal plane. The possible reactions were freezing, curling the body, and walking. Significantly different reactions were recorded upon receiving slight and strong vibration perceived on the ground (Chi-square test: *χ*^2^ = 97.5, df = 2, *P* < 0.001). In particular, Analysis of Standardized Residuals (see Supplementary Table S1) showed that the slight vibration caused a higher fraction of ants to engage in freezing (38/60) than expected (19.5/60) (std. residual: 4.2, *P* < 0.001) and walking (19/60) than expected (10.5/60) (std. residual: 2.6, *P* < 0.01), while a lower number of ants assumed a curled position (3/60) than expected (std. residual: - 4.9, 30/60) (*P* < 0.001). On the contrary, upon receiving a strong vibration, curling was adopted by a higher number of ants (57/60) than expected (30/60) (std. residual: 4.9, P < 0.001), while freezing and walking by a lower number (respectively 1/60 and 2/60) than expected (respectively 19.5/60 and 10.5/60) (std. residuals respectively: -4.2, -2.6, P < 0.001). Significant differences in the frequency of responses were also recorded upon receiving slight and strong tapping on the gaster (Chi-square test: *χ*^2^ =111.6, df = 2, P < 0.001). In the first case, a higher fraction of ants engaged in freezing (53/60) than expected (26.5/60) (std. residual: 5.1, *P* < 0.001) while walking (7/60) was not different from expected (5/60) (std. residual: 0.9, P = ns), no ant assumed a curled position (0/60) (expected 28.5/60) (std. residual: -5.3, *P* < 0.001). Upon receiving a strong vibration, curling was adopted by a higher number of ants (57/60) than expected (28.5/60) (std. residual: 5.3, *P* < 0.001), while no freezing was recorded (0/60, expected 26.5/60, std. residual: -5.1, *P* < 0.001) and only a few ants walked (3/60, expected 5/60, std. residual: -0.9, *P* = ns). To summarize, upon receiving a slight vibration or tapping, the most commonly adopted behaviour was sudden freezing, while after strong vibration or tapping (making the ants losing the contact with the substrate or bouncing), most ants assumed a curled position.


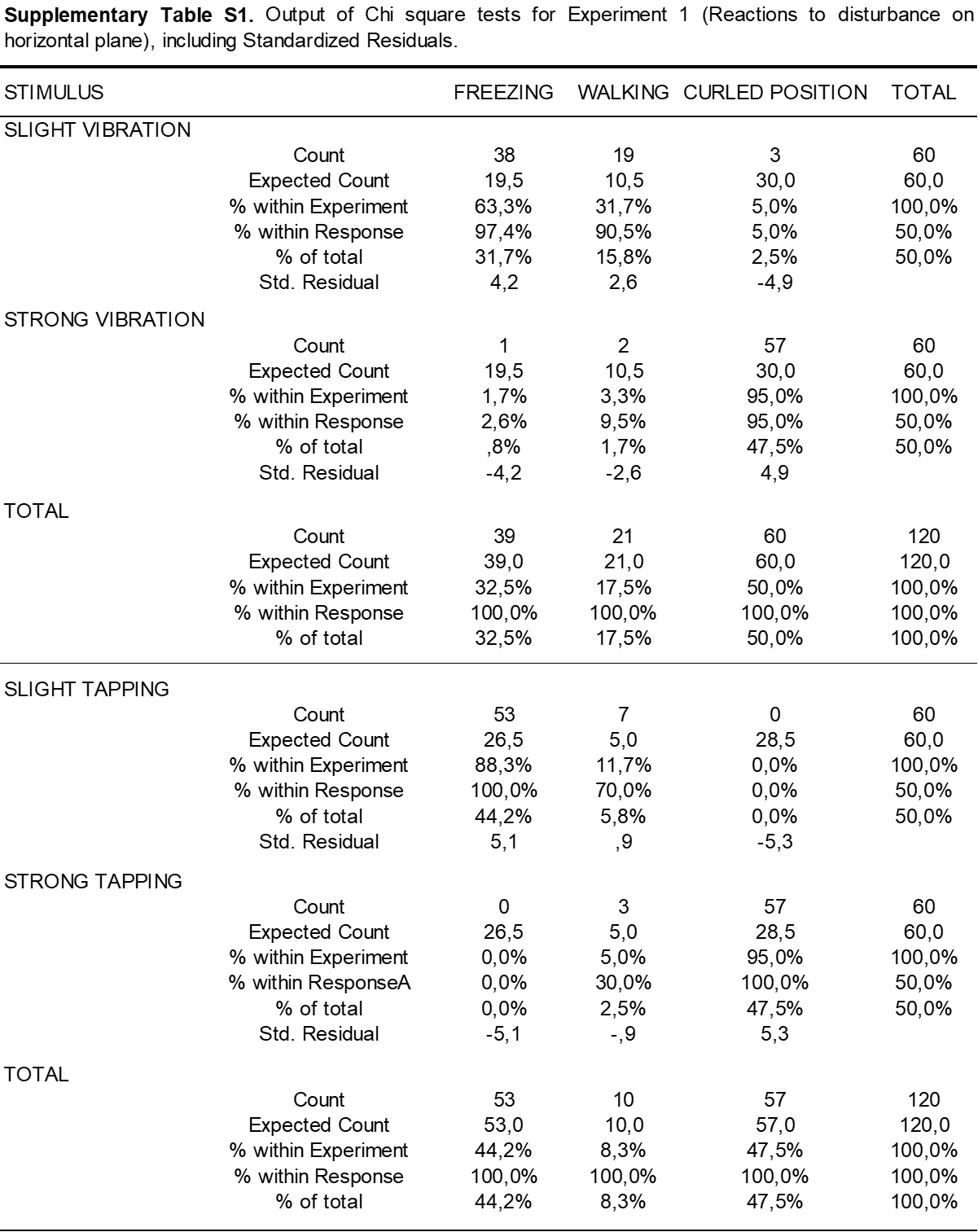


**Legend to Supplementary Videos**

**Supplementary Video 1.** **Rolling behaviour of *M. graminicola***

An overview of the rolling behaviour of *M. graminicola* upon perceiving a mechanical stimulation (rubbing) on the substrate (leaf or inclined and horizontal experimental planes) with a description of the main phases of the process.

**Supplementary Video 2. Escape after rolling**

Rapid escape of *M. graminicola* after rolling on inclined plane in search for a safe place.
